# Associations of self-reported residential noise exposure with obesity and hypertension in children and adolescents

**DOI:** 10.3389/fped.2022.902868

**Published:** 2022-08-12

**Authors:** Xiaohua Liang, Xian Tang, Mingliang Liu, Xiaoyue Liang, Li Chen, Xia Chen, Lei Zuo, Yanling Ren, Guang Hao

**Affiliations:** ^1^National Clinical Research Center for Child Health and Disorders, Ministry of Education Key Laboratory of Child Development and Disorders, Chongqing Key Laboratory of Pediatrics, Clinical Epidemiology and Biostatistics Department, Children's Hospital of Chongqing Medical University, Chongqing, China; ^2^Department of Public Health and Preventive Medicine, School of Medicine, Jinan University, Guangzhou, China; ^3^Department of Medicine, Medical College of Georgia, Georgia Prevention Institute, Augusta University, Augusta, GA, United States

**Keywords:** transportation noise, obesity, hypertension, children, mediating effect

## Abstract

**Background:**

Epidemiologic evidence linking environmental noise to obesity and hypertension remains scarce, especially in children, and the results remain inconclusive. This study aims to examine the cross-sectional associations of self-reported residential noise exposure with obesity and hypertension in children and adolescents.

**Methods:**

As an ongoing study, a representative sample of the children aged 6–9 years in Chongqing were selected in 2014. In 2019, self-reported residential noise (answer categories: “very quiet,” “moderately quiet,” “slightly quiet,” and “not at all quiet”) data were collected, and 3,412 participants with completed data were included in the analyses.

**Results:**

Participants living in a quieter area had a significantly lower risk of obesity than those living in a noisy area (very quiet: OR = 0.50, 95%CI: 0.29–0.88, *P* = 0.015; moderately quiet: OR = 0.61, 95%CI: 0.36–1.02, *P* = 0.059). Similar associations were observed for abdominal obesity, although did not reach statistical significance. Consistently, residential noise exposure was significantly associated with body mass index (BMI) and waist-to-height ratio. Self-reported residential noise exposure was positively associated with systolic blood pressure (β = −1.808; 95%CI = −3.495, −0.110; *P* = 0.037). When sleep quality, study stress, BMI, and vegetable/fruits consumption were further adjusted, all effect estimates decreased, and no statistical association was observed between noise exposure and blood pressure. Furthermore, we found that the mediating effects of obesity on the associations of self-reported residential noise exposure with hypertension were 6.8% (% of total effect mediated = 0.068, 95%CI: −2.58, 3.99), although did not reach statistical significance.

**Conclusions:**

Self-reported residential noise exposure was associated with a higher risk of obesity or abdominal obesity. Also, self-reported residential noise exposure was positively associated with hypertension, and obesity may partially mediate this association, but did not reach statistical significance.

## Introduction

Childhood obesity and hypertension continue to increase worldwide. It is estimated that 8% of children and adolescents had hypertension during 2010-14, and 6% of girls and 8% of boys were obese in 2016 (1, 2). Childhood obesity and hypertension contribute to many chronic diseases in adults, such as heart disease and kidney disease. (3–5) The increase in health risks and socioeconomic losses related to obesity and hypertension have aroused widespread concern (6). Therefore, identifying early modifiable factors for preventing childhood obesity and hypertension is required.

Poor dietary quality and sedentary lifestyles have been known to cause obesity and hypertension (7, 8). In recent decades, environmental noise has been attracting growing attention. Previous studies have investigated the associations of environmental noise with the risk of obesity and hypertension in children or adults, but the results are controversial and the effect size was small even though a positive association was observed (9–13). It is suggested that noise sensitivity, rather than the noise level, predicts the non-auditory health hazards of noise (14). However, almost all these studies adopted model estimated road traffic noise in the analysis, which may be inaccurate as the layout of rooms in the house, window opening habits, and indoor noise levels were not taken into account (15), indeed, noise sensitivity was also not considered. Also, most of these findings come from Europe and North American countries. In this study, we examined the cross-sectional associations of self-reported residential noise exposure with obesity and hypertension among Chinese children and adolescents.

## Methods

In 2014, a stratified cluster sampling was used to obtain a representative sample of the children aged 6–9 years in Chongqing (16). The first stage of sampling was to randomly select one rural and one urban county, then two communities per county were randomly selected. Participants were recruited if they: (1) were aged between six and nine years in 2014, (2) resided in the target region for more than 6 months, (3) did not have serious diseases (e.g., nephropathy, cardiovascular disease, or cancer), and obtained consent both from the parents and children for participation. A total of 5,246 children living in the selected communities were informed and included if they satisfied the inclusion criteria. At baseline, demographic information and physical examination data were collected. As an ongoing study, 4,162 participants were followed up in 2019. Besides the physical examination, self-reported residential noise was collected. In this study, 3,412 participants with completed data in 2019 were included in the analyses. All work in this study was conducted following the ethical guidelines of the 1964 Declaration of Helsinki and its later amendments. The Institutional Review Board at the Children's Hospital of Chongqing Medical University approved the study (File No.: 2019–86). Informed consent was obtained from all participants and their parents/guardians.

### Measurements and definitions

Anthropometric measurements were performed by well-trained pediatric nurses as described in a previous publication. (16) A mobile medical ultrasonic machine (models-H300D) was used to measure height and weight, and body mass index (BMI) was calculated as weight/height^2^ (kg/m^2^). Obesity was defined according to Chinese guidelines for children and adolescents (Supplementary Table 1) (17). Waist circumstance (WC) (18) was measured twice at the center of the umbilicus over one T-shirt and the values were averaged. The waist-to-height ratio (WHtR) was calculated as WC divided by the height. The cut-off value of 0.48 was used for WHtR to classify abdominal obesity (19).

Blood pressure (BP) was measured using an OMRON arm-type electronic sphygmomanometer (HEM7051) (16). BP measurements were taken at 11, 13, and 15 min after a 10-min seated rest in the morning (09:00–12:00) using a proper sized cuff on the right arm. The average of the three measurements was taken to represent resting systolic blood pressure (SBP) and diastolic blood pressure (DBP) levels. Hypertension was defined as a mean SBP and/or DBP ≥95th percentile (20).

Residential noise was assessed by the question “is your flat or house quiet (answer categories: ‘very quiet', ‘moderately quiet', ‘slightly quiet', and ‘not at all')?”

Demographic information was collected by a standardized questionnaire. Study stress was assessed by the question ‘Do you feel study stress over last year 1) extremely, 2) very, 3) moderately, 4) slightly, or 5) no at all.' The participants answered 1) extremely, 2) very, or 3) moderately were defined as having study stress. A previously reported quantitative food frequency questionnaire (FFQ) was used to collect dietary information (21). The questionnaire was filled by the parents or guardians of the children after standard training by the research team. The Children's Sleep Habits Questionnaire (CSHQ) was used to assess the children's sleep quality (22). A score 1 to 3 was assigned to each response. The overall score ranged from 0 to 99 by summarizing the scores of all 33 items. A higher score indicated a worse sleep quality. The exercise was assessed by the self-reported vigorous/moderate physical activity (including playing basketball, swimming, hiking, running, riding a bike, etc.). The number of minutes per day was calculated (16).

### Statistical analyses

Differences of continuous variables in general characteristics of participants were assessed using a *t*-test, and a χ^2^ test was used to compare the difference of categorical variables. Logistic regression was used to analyze the associations of residential noise exposure with obesity, abdominal obesity, and hypertension, while a linear regression model was performed to test the associations of self-reported residential noise exposure with BMI, WHtR, SBP, and DBP. For estimating the direct effect and total effect of self-reported noise exposure on outcomes, a Directed Acyclic Graph (DAG) was constructed to represent plausible causal assumptions in the context of possible confounding factors and intermediate pathways (Supplementary Figure 1) (23). For obesity, univariate analyses were performed in Model 1. Model 2 was adjusted for age, sex, exercise, father's education, and region (urban/rural). Model 3 additionally controlled for sleep quality, study stress, and vegetable/fruits consumption. For hypertension, BMI was further adjusted in model 3. Also, the mediation effect of BMI on the association between self-reported residential noise exposure and hypertension (“medeff” package). All statistical analyses were implemented using Stata software version 12 (STATA Corp., TX, US). A two-sided *P* < 0.05 was considered statistically significant.

## Results

The general characteristics of the participants are presented in Table 1. A total of 3,412 participants were included, who were aged 11.2 years on average, and 49.1% were boys. About 50% of participants were living in very quiet or moderately quiet areas.

**Table 1:** Characteristics of participants by gender.

	**Girls**	**Boys**	***P-*value**
Age(years)	11.2 ± 0.0	11.2 ± 0.0	0.008
Height (cm)	151.7 ± 0.2	151 ± 0.2	0.012
Weight (kg)	43.2 ± 0.2	43.8 ± 0.3	0.065
Exercise (min/d)	98.7 ± 4.2	157.0 ± 7.0	<0.001
Sleep quality	49.1 ± 0.4	49.0 ± 0.4	0.790
Vegetable/fruit (g/d)	331.2 ± 19.6	312.1 ± 33.4	0.624
Father's education ^*^ (*n*, %)	33.1(30.9–35.4)	34.6(32.4–36.9)	0.341
Study stress (*n*, %)	25.4(23.4–27.6)	23.6(21.7–25.6)	0.207
Urban (*n*, %)	42.7(40.3–45.0)	42.0(39.7–44.3)	0.701
Self-reported noise exposure (*n*, %)			
Not at all quiet	59(3.5)	65(3.7)	0.124
Slightly quiet	453(27.1)	424(24.4)	
Moderately quiet	774(46.2)	793(45.6)	
Very quiet	388(23.2)	456(26.2)	

* *Father's education >12 years. Data were presented as mean with standard error or percentage (95%CI)*.

**Figure 1:**
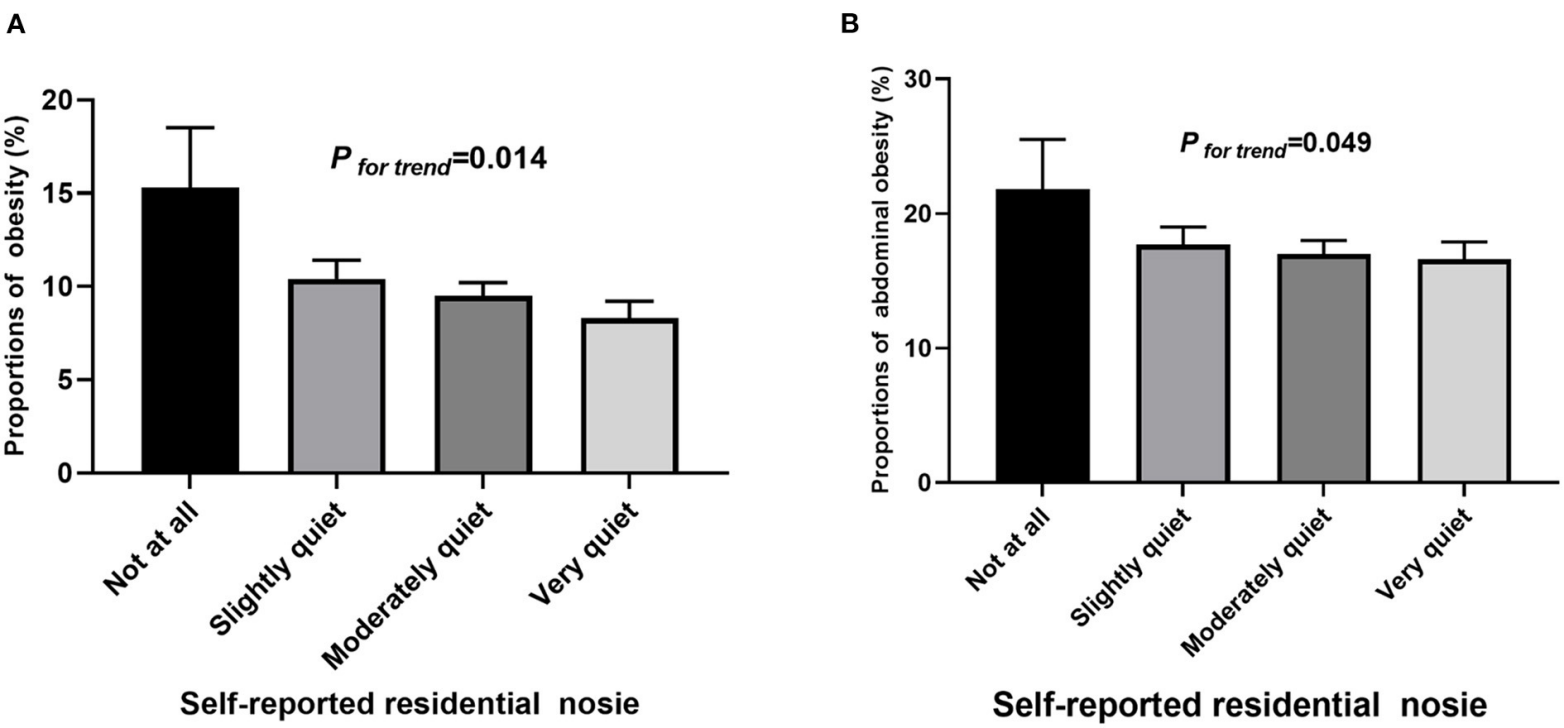
Proportions of **(A)** obesity and **(B)** abdominal obesity by self-reported residential noise exposure.

The proportions of obesity were 15.3, 10.4, 9.5, and 8.3% in participants living in not at all, slightly, moderately and very quiet areas, while these proportions for abdominal obesity were 21.8, 17.7, 17.0, and 16.6%, respectively (Figure 1). The participants living in a quieter area had a significantly lower risk of obesity than those living in a noisy area (very quiet: OR = 0.50, 95%CI: 0.29–0.88, *P* = 0.015; moderately quiet: OR = 0.61, 95%CI: 0.36–1.02, *P* = 0.059). Model 3 further controlled for the sleep quality, study stress, and vegetable/fruits consumption, the results remain similar (very quiet: OR = 0.49, 95%CI: 0.28–0.86, *P* = 0.012; moderately quiet: OR = 0.59, 95%CI: 0.35–0.99, *P* = 0.047). Similar associations were observed for abdominal obesity, although did not reach statistical significance (Table 2). Consistently, residential noise exposure was significantly associated with BMI and WHtR (Table 3).

**Table 2:** Associations of self-reported noise exposure with obesity and abdominal obesity.

**Residential noise**	**Obesity**			**Abdominal obesity**		
	**OR**	**95%CI**	***P*-value**	**OR**	**95%CI**	***P*-value**
Model 1						
Not at all quiet	Reference			Reference		
Slightly quiet	0.64	0.37, 1.09	0.102	0.77	0.49, 1.22	0.265
Moderately quiet	0.58	0.35, 0.97	0.039	0.74	0.47, 1.15	0.183
Very quiet	0.50	0.29, 0.86	0.013	0.77	0.49, 1.22	0.265
Model 2						
Not at all quiet	Reference			Reference		
Slightly quiet	0.67	0.39, 1.14	0.140	0.79	0.50, 1.27	0.335
Moderately quiet	0.61	0.36, 1.02	0.059	0.72	0.46, 1.14	0.165
Very quiet	0.50	0.29, 0.88	0.015	0.66	0.41, 1.07	0.091
Model 3						
Not at all quiet	Reference			Reference		
Slightly quiet	0.65	0.38, 1.12	0.124	0.77	0.48, 1.24	0.281
Moderately quiet	0.59	0.35, 0.99	0.047	0.70	0.44, 1.11	0.130
Very quiet	0.49	0.28, 0.86	0.012	0.64	0.40, 1.03	0.067

*OR, odds ratio; CI, confidence interval. Model 1, unadjusted; Model 2, adjusted for age, sex, exercise, father's education, and region (urban/rural); Model 3, Model 2+ sleep quality, study stress, and vegetable/fruits consumption*.

**Table 3:** Associations of self-reported noise exposure with body mass index and waist-to-height ratio.

**Residential noise**	**Body mass index**			**Waist–to–height ratio**		
	**β**	**95%CI**	***P*–value**	**β**	**95%CI**	***P*–value**
Very quiet	Reference			Reference		
Moderately quiet	−0.669	−1.326, −0.010	0.046	−0.009	–.020, 0.000	0.092
Slightly quiet	−0.810	−1.449, −0.170	0.013	−0.012	–.022, 0.000	0.029
Not at all quiet	−0.813	−1.471, −0.150	0.016	−0.014	–.024, 0.000	0.013

*OR, odds ratio; CI, confidence interval. Adjusted for age, sex, region (urban/rural), exercise, and father's education*.

**Figure 2:**
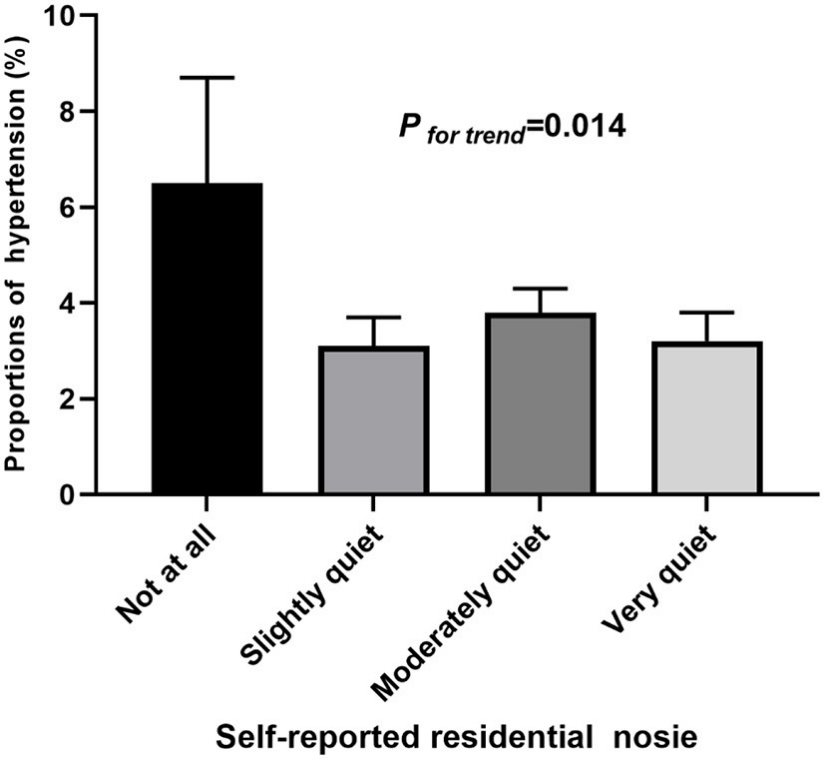
Proportions of hypertension by self-reported residential noise exposure.

The proportions of hypertension were 6.5, 3.1, 3.8, and 3.2% in participants living in not at all, slightly, moderately, and very quiet areas (Figure 2). Self-reported residential noise exposure was positively associated with hypertension and SBP after adjusting for age, sex, exercise, father's education, and region (urban/rural) (β = −1.808; 95%CI = −3.495, −0.110; *P* = 0.037) in model 2. After further adjusting for sleep quality, study stress, BMI, and vegetable/fruits consumption, all effect estimates decreased, and no statistical association was observed (Table 4). Furthermore, we found that the mediating effect of obesity on the associations of self-reported residential noise exposure with hypertension was 6.8% (% of total effect mediated = 0.068, 95%CI: −2.58, 3.99), although failed to achieve statistical significance.

**Table 4:** Associations of self–reported noise exposure with hypertension and blood pressure.

**Residential noise**	**Hypertension**			**Systolic blood pressure**			**Diastolic blood pressure**		
	**OR**	**95%CI**	***P*-value**	**β**	**95%CI**	***P*-value**	**β**	**95%CI**	***P*-value**
Model 1									
Not at all quiet	Reference			Reference			Reference		
Slightly quiet	0.46	0.20, 1.03	0.060	−1.877	−3.583, −0.171	0.031	−0.420	−1.676, 0.837	0.513
Moderately quiet	0.56	0.26, 1.19	0.131	−1.485	−3.144, 0.174	0.079	−0.070	−1.292, 1.153	0.911
Very quiet	0.47	0.21, 1.05	0.067	−1.310	−3.020, 0.400	0.133	0.091	−1.168, 1.350	0.887
Model 2									
Not at all quiet	Reference			Reference					
Slightly quiet	0.46	0.20, 1.05	0.066	−1.803	−3.495, −0.110	0.037	−0.335	−1.581, 0.910	0.598
Moderately quiet	0.61	0.28, 1.31	0.204	−1.206	−2.853, 0.440	0.151	0.171	−1.041, 1.380	0.782
Very quiet	0.53	0.23, 1.20	0.125	−0.968	−2.666, 0.730	0.264	0.365	−0.885, 1.620	0.567
Model 3									
Not at all quiet	Reference			Reference			Reference		
Slightly quiet	0.57	0.24, 1.38	0.214	−1.071	−2.592, 0.450	0.168	−0.036	−1.245, 1.174	0.954
Moderately quiet	0.77	0.33, 1.77	0.535	−0.322	−1.807, 1.162	0.670	0.529	−0.651, 1.710	0.379
Very quiet	0.68	0.28, 1.66	0.403	−0.082	−1.611, 1.447	0.916	0.725	−0.490, 1.941	0.242

*OR, odds ratio; CI, confidence interval. Model 1, unadjusted; Model 2, adjusted for age, sex, exercise, father's education, and region (urban/rural); Model 3, Model 2+ sleep quality, study stress, body mass index, and vegetable/fruits consumption*.

## Discussions

We found that self-reported residential noise exposure was significantly associated with a higher risk of obesity and abdominal obesity. Also, self-reported residential noise exposure was positively associated with hypertension, and obesity may partially mediate this association, although these findings did not reach statistical significance.

Several studies, most performed in European countries, examined the associations between transportation noise exposure and obesity or its markers in adults using cross-sectional and longitudinal designs. Overall, current results are mixed (24–27), and the magnitude was small even if a significant association was reported (25, 28). Few studies investigated whether transportation noise is related to obesity and its markers in children and adolescents, and the results were also inconsistent (29). The Danish National Birth Cohort reported marginally significant associations between residential road traffic noise exposure during pregnancy and childhood and risk for childhood overweight and statistically non-significant associations for BMI z-scores (30). However, another longitudinal study reported that BMI curves from birth to 8 years were only associated with road traffic noise exposure during pregnancy, but not with the exposure during childhood (31). A Norwegian study observed associations with both adipose markers only in highly noise-sensitive women (14). In line with this study to some extent, we found a significant association between self-reported residential noise exposure and obesity with a large effect size in children and adolescents.

Epidemiologic evidence linking environmental noise to hypertension also remains scarce, especially in children, and the results remain inconclusive. In a large case-control study, Zeeb et al. found no association between residential traffic noise exposure and hypertension in the primary analysis, but a significant positive association in persons with an initial hypertension diagnosis (32). A study from Canada reported that long-term exposure to road traffic noise was longitudinally associated with an increased risk of hypertension in adults (33). A research conducted among Indian adult population reported that the exposure to road traffic noise at L den > 65 dB(A) in male and L den > 60 dB(A) in female was correlated to the prevalence of hypertension (34). A cross-sectional study involving 500 Chinese adults found that indoor nocturnal noise was associated with BMI and BP in females but only BP in males (35). One possible explanation is that females might be more annoyed by noise than males (36). To our best knowledge, this study, for the first time, reported the association between self-reported residential noise and hypertension in children and adolescents, and the results suggested that obesity may partially mediate this association.

The mechanisms underlying the associations of environmental noise and obesity and hypertension are not fully understood. Multiple health conditions, physically or mentally, have been linked to the environmental noise exposure, which includes sleep disorders (37, 38). Noise-induced sleep disorders may play a role in these associations. Children's sleep duration is negatively associated with overweight in both cross-sectional and longitudinal studies, possibly through the influence on insulin resistance, sedentarism, and unhealthy dietary patterns (39–41). On the other hand, children with obstructive sleep apnea have increased sympathetic activation during sleep, blunted dipping, or elevated systolic or diastolic pressures (42). Moreover, traffic noise exposure has been suggested to trigger stress in children in previous studies (43). Stress caused by exposure to environmental noise could be increasing the risk of obesity and hypertension (44, 45).

## Limitations and strengths

One major strength was that we investigated the associations of residential noise exposure with obesity and hypertension in children and adolescents using self-reported residential noise data, to some extent, which may be a better index taking account of noise sensitivity at the same time to test the effect of residential noise on health. There were also several limitations in our study. First, exposure to noise at school may have a more substantial effect on BP in children (46), but we did not account for exposure outside of the home in this analysis. Second, this study cannot infer causality due to the cross-sectional study design. Moreover, estimating mediation analysis with cross-sectional data will overestimate or underestimate the mediation effect. Third, we could not include all risk factors due to the limitation of the data, such as genotypes, air pollution et al. More cohort studies, including objective and individual level environmental noise measurement, are desirable to confirm the results. Fourthly, the father's education may not the best indicator of the family's socioeconomic status. Finally, the noise level was divided into four categories by participants' subjective feelings, so future studies considering the objective measurement of noise exposure using a noise meter and subjective measurement of noise sensitivity using standardized questionnaire are required.

## Conclusion

In conclusion, this study showed that self-reported residential noise exposure was associated with a higher risk of obesity and abdominal obesity. Also, self-reported residential noise exposure was positively associated with the risk of hypertension, and obesity may partially mediate this association, although these findings did not reach statistical significance. Further studies will be necessary to confirm our results.

## Data availability statement

The raw data supporting the conclusions of this article will be made available by the authors, without undue reservation.

## Ethics statement

The studies involving human participants were reviewed and approved by Institutional Review Board at the Children's Hospital of Chongqing Medical University. Written informed consent to participate in this study was provided by the participants' legal guardian/next of kin.

## Author contributions

GH conceived and designed the study. XT, XHL, and YR collected the data. XHL contributed to the performance of the statistical analysis and interpretation of the results and wrote the first draft of this manuscript. ML, XC, LZ. XT, XYL, LC, and YR critically reviewed and approved the final paper. All authors contributed to the article and approved the submitted version.

## Funding

This work was supported by the Basic Research Project of Key Laboratory of Ministry of Education of China in 2021(GBRP-202106), Major Health Project of Chongqing Science and Technology Bureau (No. CSTC2021jscx-gksb-N0001), Research and Innovation Team of Chongqing Medical University (No. W0088), Joint Medical Research Project of Chongqing Municipal Health Commission and Chongqing Science and Technology Bureau (No. 2020MSXM062), National Key Research and Development Project (2017YFC0211705), Education Commission of Chongqing Municipality (KJQN201900443) and National Natural Science Foundation of China (82003521, 81502826). The research is also financially supported by Hunan Provincial Key Laboratory of Clinical Epidemiology (2021ZNDXLCL002).

## Conflict of interest

The authors declare that the research was conducted in the absence of any commercial or financial relationships that could be construed as a potential conflict of interest.

## Publisher's note

All claims expressed in this article are solely those of the authors and do not necessarily represent those of their affiliated organizations, or those of the publisher, the editors and the reviewers. Any product that may be evaluated in this article, or claim that may be made by its manufacturer, is not guaranteed or endorsed by the publisher.
