# Supplementary material for: Associations of self-reported residential noise exposure with obesity and hypertension in children and adolescents
Source: Front Pediatr. 2022 Aug 12;10:902868. doi: 10.3389/fped.2022.902868 (PMC9411713; doi:10.3389/fped.2022.902868)
Supplement: Supplementary file 1 [file Data_Sheet_1.docx]

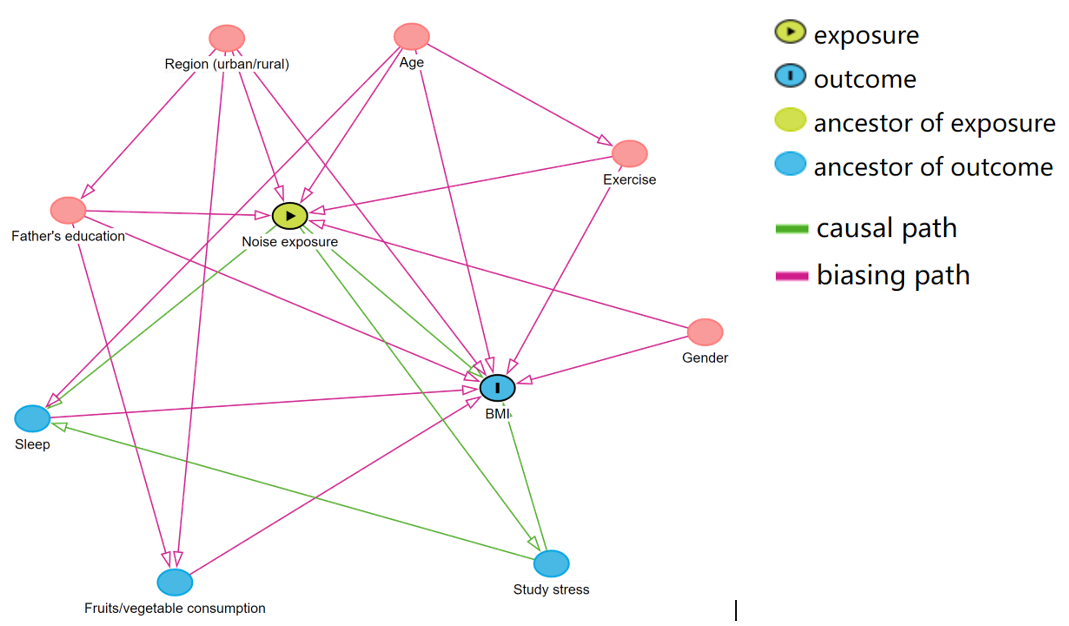
SFigure 1. Directed acyclic graph describing the potential association between self-reported noise exposure and body mass index. DAGitty version 3.0.

Red color variables were adjusted for in the Model 2, and all variables were adjusted for in Model 3.

Stable1 Obesity definitions by body mass index according to the Chinese guidelines for children and adolescents

| Age | Boys | Girls |
| --- | --- | --- |
| 7~ | 19.2 | 18.9 |
| 8~ | 20.3 | 19.9 |
| 9~ | 21.4 | 21.0 |
| 10~ | 22.5 | 22.1 |
| 11~ | 23.6 | 23.3 |
| 12~ | 24.7 | 24.5 |
| 13~ | 25.7 | 25.6 |
| 14~ | 26.4 | 26.3 |
| 15~ | 26.9 | 26.9 |
| 16~ | 27.4 | 27.4 |
| 17~ | 27.8 | 27.7 |
| 18 | 28.0 | 28.0 |
